# Beyond Cholecystitis: Unveiling Dorsal Pancreatic Agenesis in Routine Imaging

**DOI:** 10.7759/cureus.83225

**Published:** 2025-04-29

**Authors:** Jairo Alejandro Otero Coral, Valentina Rincón Giraldo, Lizbelky Cristina Mora Tous, Julián Andrés Muñoz Durán

**Affiliations:** 1 Emergency Medicine, Clínica Panamericana, Apartadó, COL; 2 Radiology, Clínica Soma, Medellin, COL; 3 Interventional Radiology, Universidad de Antioquia, Medellin, COL

**Keywords:** atrophy of the pancreas, computed tomography abdomen, incidental radiological finding, laboratories, non specific abdominal pain

## Abstract

Dorsal pancreatic agenesis (DPA) is a rare congenital disorder resulting from abnormal embryogenesis, where the body and tail of the pancreas are absent while the head remains intact. This case report describes a 21-year-old male presenting with symptoms suggestive of acute cholecystitis. Diagnostic imaging, including abdominal ultrasound and contrast-enhanced computed tomography, confirmed acute cholecystitis and incidentally identified complete dorsal pancreas agenesis. The patient underwent laparoscopic cholecystectomy and was discharged asymptomatic after 72 hours for outpatient follow-up. Despite its rarity, dorsal pancreas agenesis is important to recognize, as it can be asymptomatic and often discovered incidentally during evaluations for other conditions. Understanding the embryological development of the pancreas, the clinical implications of dorsal pancreas agenesis, and its potential association with diabetes mellitus is crucial for healthcare professionals. This case underscores the need for meticulous interpretation of imaging studies and highlights the importance of considering anatomical variants in the differential diagnosis of abdominal pain.

## Introduction

Dorsal pancreatic agenesis (DPA) is the anatomical absence of the body and tail of the pancreas. It is usually clinically asymptomatic but may be associated with diabetes mellitus, abdominal pain, and non-alcoholic pancreatitis. It is a rare congenital disorder characterized by genetic alterations that lead to abnormal formation during embryogenesis; however, it is considered compatible with life, unlike complete agenesis or ventral agenesis of the pancreas, which are incompatible with life [1]. From the fourth week of gestation, the primitive intestine is closed, and it is from this structure that the organs and tissues of the digestive system originate. The primitive intestine is divided into three segments: anterior, middle, and posterior. The pancreas originates from the caudal portion of the anterior intestine, where endodermal cells proliferate, forming two buds, namely ventral and dorsal. The ventral pancreatic bud gives rise to the lower part of the pancreatic head, the uncinate process, and the anterior part of the pancreatic duct. The dorsal bud gives rise to the upper part of the pancreatic head, the body, and the tail. The head is essential for the exocrine function of the pancreas, while the endocrine function is directly related to the pancreatic tissue [1,2].

## Case presentation

A 21-year-old man with no significant past medical history, including no history of diabetes or other chronic conditions, and no relevant family medical history, presented to the emergency room with severe pain in the right upper quadrant of the abdomen that had been ongoing for 24 hours, associated with nausea and vomiting. On physical examination, he exhibited tenderness in the right upper quadrant, a positive Murphy's sign, and a fever of 38.5°C, initially suggestive of acute cholecystitis. Laboratory tests revealed leukocytosis (white blood cell count: 15,800/µL; normal range: 4,500-11,000/µL), hyperbilirubinemia (direct bilirubin: 1 mg/dL, indirect bilirubin: 2 mg/dL), and a non-fasting blood glucose level of 101 mg/dL (normal range: less than 140 mg/dL) (Table 1).

**Table 1:** Laboratory test results of the patient

Laboratory test	Patient value	Normal range
Hemoglobin	13.2 g/dL	13.5-18 g/dL
Hematocrit	41%	40-54%
White blood cells	15,800/µL	4,500-11,000/µL
Neutrophils	86%	40.3-74.8%
Lymphocytes	12.5%	12.2-47.1%
Monocytes	1%	4.4-12.3%
Direct bilirubin	1 mg/dL	0-0.5 mg/dL
Indirect bilirubin	2 mg/dL	0.2-1.2 mg/dL

Due to the high clinical suspicion of acute cholecystitis, an abdominal ultrasound was performed, which confirmed the presence of cholelithiasis and inflammatory signs of the gallbladder. However, during the ultrasound study, the pancreas was difficult to visualize adequately. For this reason, an abdominal contrast-enhanced computed tomography (CECT) was requested.

**Figure 1:**
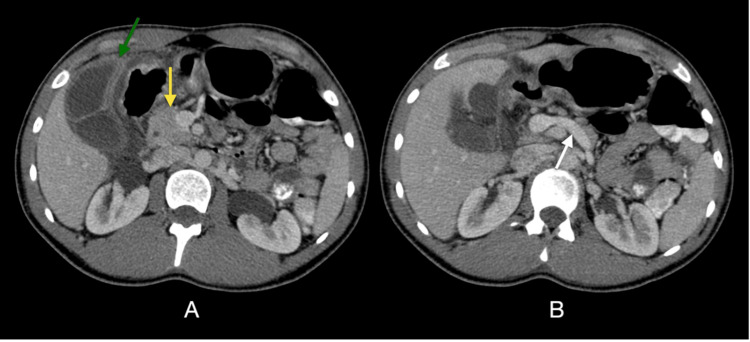
Acute cholecystitis in a case of complete agenesis of the dorsal pancreas Axial contrast-enhanced computed tomography (CECT) shows (A) a normal-appearing pancreatic head (yellow arrow), but neither body nor tail is present. This patient has a complete congenital absence of the dorsal pancreas and has additional gallbladder wall thickening (>3 mm) with mural/mucosal hyperenhancement for acute cholecystitis (green arrow). In (B), no pancreatic tissue is in the body-tail segments' expected position. There should normally be pancreatic tissue along the anterior aspect of the splenic vein (white arrow).

The CT scan (Figure 1) confirmed the findings of acute cholecystitis but incidentally also revealed the complete absence of the body and tail of the pancreas, with only the head portion being visualized, which was compatible with congenital dorsal pancreas agenesis. No other associated abnormalities were observed.

The patient underwent laparoscopic cholecystectomy and was discharged asymptomatic after 72 hours for outpatient follow-up care.

## Discussion

Embryologically, the pancreas develops from endodermal tissue from the fifth week of gestation. Its head and uncinate process arise from the ventral portion. The dorsal portion forms the neck, body, and tail, and by the seventh week of gestation, the ventral portion rotates and fuses with the dorsal portion. Subsequently, the Wirsung duct and its papilla are formed from the ventral portion after this fusion. The head of the pancreas is the essential anatomical structure for the exocrine function of the pancreas. It is responsible for producing digestive enzymes (amylase, lipase, and proteases) that are important for the digestion of different food groups. At the same time, the minor duct or accessory duct (Santorini) originates from the neck, body, and tail (dorsal pancreas) [1,2].

DPA was first described in 1911 by Heigberg during an autopsy as an incidental finding. Approximately 100 cases have been published, of which 22 are pediatric patients. DPA is presumed to be caused by defects in embryogenesis [3-5]. Although no exact genetic cause has been established, a close correlation between the hepatocyte nuclear factor 1 beta (HNF1-β) and GATA binding protein 6 genes and the presentation of DPA has been reported. Experimental studies in rodents have shown that the mutation of Raldh2 and the H1x9b gene, or retinoic acid deficiency, can lead to the development of DPA [5-7]. There is insufficient evidence to demonstrate an underlying family history; however, a case report documented the presence of DPA and diabetes mellitus in a woman and her two children, suggesting that it could be an autosomal dominant or X-linked disease [8].

DPA can present as partial or total, depending on the absence or presence of the pancreatic tail, respectively. The incidence of symptoms is variable, regardless of the type of presentation. The most frequently related symptoms are abdominal pain, non-alcoholic pancreatitis, and diabetes mellitus. The latter is associated with the presentation of total DPA; most pancreatic islets are located in the tail, leading to greater insulin deficiency [4,5,8].

Symptoms are nonspecific, and diagnosis usually occurs as an incidental finding during the evaluation of another unrelated pathology or in the search for the cause of abdominal pain. Diagnosis via ultrasound is difficult due to the interposition of intestinal gas; more useful methods include contrast-enhanced abdominal computed tomography or magnetic resonance imaging (MRI). Differential diagnoses include pancreatic fat infiltration, chronic pancreatitis, and atrophy of the pancreatic tail [7,9]. The main risk associated with DPA is the development of diabetes mellitus due to the deficiency in insulin production, as a strong association has been demonstrated in reported cases [3,4].

DPA does not require specific treatment; instead, the associated conditions that may arise, such as diabetes, will be managed with exogenous insulin supplementation.

A strong component of this case report is the incidental discovery of complete DPA during the workup of acute cholecystitis which underscores the importance of thorough radiologic assessment during clinical practice. The clear imaging documentation and incorporation of the embryological rationale enhance its didactic value to both the radiologist and the clinician. Still, a shortcoming of the report is a lack of long-term follow-up to evaluate the possible metabolic consequences of sustaining complete DPA which is known to lead to diabetes mellitus. Notwithstanding, this report contributes to the literature by emphasizing the need to understand the anatomical variations of the pancreas in younger patients presenting with vague abdominal pain and supporting the use of cross-sectional imaging to identify unusual congenital anomalies.

## Conclusions

Most cases of DPA are asymptomatic, and their discovery often corresponds to incidental findings in studies of other pathologies, as in our case of acute cholecystitis, where a radiologist's expertise suspected a possible anatomical variant, confirmed by tomography. This highlights the importance of accurate reading and interpretation of diagnostic aids and interdisciplinary management, allowing for a broader approach in such cases. There are few studies on this variant; however, it is known that diabetes mellitus is strongly associated with complete dorsal agenesis, especially in young patients, and early insulin initiation warrants healthcare professionals to consider this anatomical variant as a possible etiology of the disease.
